# The effects of anaesthesia on the developing brain: a summary of the clinical evidence

**DOI:** 10.12688/f1000research.2-166.v2

**Published:** 2013-08-20

**Authors:** Clara KY Yu, Vivian Man Ying Yuen, Gordon TC Wong, Michael G Irwin

**Affiliations:** 1 Department of Anaesthesiology, Queen Mary Hospital, Hong Kong; 2 Department of Anaesthesiology, University of Hong Kong, Hong Kong

## Abstract

**Introduction**: There is data amassing in the literature regarding the potentially adverse effects of anaesthesia exposure on the developing human brain. The purpose of this article is to summarise current relevant data from clinical studies in this area.

**Methods**: Articles from journals written in English were searched for using PubMed, Ovid and Medline. Keywords used included: brain (newborn, infant, child and neonate), neurodegeneration, apoptosis, toxicity, neurocognitive impairment (developmental impairment and learning disorders) and anaesthesia (intravenous, inhalational and sedation).

**Results**: From the initial search, 23 articles were identified as potentially relevant, with publication dates spanning from 1978 to 2012.  Twelve studies were deemed irrelevant to the research questions. The results of neurocognitive assessment from eight of the remaining eleven studies had showed some differences in the performances of children exposed to anaesthesia. The control population in these studies was highly variable. The age at which the subjects were exposed to anaesthesia ranged from prenatal to 4 years in the majority of studies with one including children aged up to 12 years when exposed.

**Discussion**: Although there is clinical data suggesting a possible detrimental effect, the evidence is best considered preliminary and inconclusive at this stage. Many of the outcome measures were lacking in specificity and standardization in most cases. Parents should be counselled to not avoid necessary invasive procedures for fear of a currently ill-defined risk.  However, deferral of elective procedures beyond the first few years of life should be contemplated.

## Introduction

Advances in perioperative care and imaging have resulted in more neonates, infants and children undergoing procedures requiring anaesthesia. General anaesthesia is a incompletely understood, complex pharmacological response produced by a heterogeneous class of drugs involving mechanisms on specific neuronal networks in different regions of the central nervous system. It is well known that the use of a balanced anaesthetic technique is beneficial in decreasing the neuroendocrine and metabolic stress response to surgery and altering pain processing ^1– 4^. However, increasing data from animal studies in the last decade has shown that general anaesthesia may also trigger apoptosis in the developing brain and suggest anaesthetic interactions with neurodegenerative mechanisms, including those linked to the onset and progression of Alzheimer's disease ^5, 6^. Naturally this has raised much concern regarding the safety of general anaesthesia in infants and young children. The developing brain differs from the adult brain in several different ways, which may provide a physiological basis for any enhanced vulnerability to anaesthetics. For example, the number of neurones formed in early development is significantly greater than in adult mammals, before synapses are pruned to establish behaviourally relevant connections between neurones. Apoptosis is responsible for eliminating 50–70% of developing neurones under normal circumstances and general anaesthesia-triggered apoptosis may disrupt this normal pattern of neural pruning ^7^. The purpose of this article is to summarise the current literature concerning the effects of anaesthesia on the developing brain and to evaluated whether the available animal data can be translated to a clinical setting. The aim is to provide up-to-date information to non-anaesthetists who may need to counsel parents in the preoperative setting.

## Method

Search engines used included PubMed, Ovid Medline and Embase, which were accessed in March 2013. Keywords used included: brain (newborn, infant, child or neonate), neurodegeneration, apoptosis, toxicity, neurocognitive impairment (developmental impairment or learning disorders) and an(a)esthesia (intravenous, inhalational and sedation). These were used in combination such that terms related to anaesthesia and brain was used together and were paired with the remaining terms in turn. Exclusion criteria were animal studies investigating anaesthesia-induced brain structural or behavioural abnormalities and articles published in non-English language journals.

## Results

A total of 23 articles were identified using the above search methods. Of these 12 were deemed irrelevant to our research question and were excluded in our discussion ( Table 1). Eight of these studies focused mainly on surgical diseases, their management and neurological outcome. The study groups were sick neonates and a majority had very low birth-weights, which adds to the multiple confounding factors. These studies were not designed to investigate anaesthetic exposure and its potential neurotoxicity. The anaesthetic technique and agent used were not specified and subsequent surgery requiring anaesthesia is unknown, hence they are excluded in our review. Three of the 12 studies were performed in the third trimester of pregnancy and one during the perinatal period. Because outcome measure of behaviour alterations was performed in the first few days after birth, any positive finding may be subtle and not relevant in the long-term outcome. Hence these studies were also excluded. Therefore, only 11 relevant publications on neurodevelopmental risk and anaesthesia exposure in early childhood were identified ^7– 16^, all of which were retrospective in nature ( Table 2). Owing to the nature of the study question, the vast majority of the studies were either cohort or case control studies. The respective control population in these studies was, however, highly variable. In all but three studies, the results of neurocognitive assessment had showed some abnormalities in the performance of children exposed to anaesthesia. The age at which the subjects were exposed to anaesthesia ranged from prenatal to 3 years in the majority of studies with one including children aged up to 12 years when exposed.

**Table 1.** List of retrieved but excluded studies.

Reference	Year of birth cohort	Study group	Control group	Age during exposure	Age during neurological assessment	Neurological sequelae in the study group
Kabra *et al.* 2007	1996–1998	PDA ligation (N=95)	Indomethacin treatment (N=245)	84% neonatal (25–29wk PCA), ELBW	18mo	Increase in cerebral palsy, cognitive delay, hearing loss, bilateral blindness
Hintz *et al.* 2005	1995–1998	Laparotomy (N=124)	Peritoneal drain placement (N=121)	Neonatal, ELBW	18–22mo	Higher frequency of cerebral palsy, lower Bayley Scales of Infant Development; no difference between medically treated patients with or without NEC
Tobiansky *et al.* 1995		NEC requiring laparotomy (N=20)	No NEC or NEC managed medically (N=40)	26–27wk PCA, VLBW	12mo, 3yr, and 5yr PCA	Higher incidence of neurodevelopmental impairment
Blakely *et al.* 2006	2001–2002	Laparotomy (N=76)	Peritoneal drain placement (N=80)	29wk PCA, ELBW	18–22mo post term	Less neurodevelopmental impairment and lower mortality
Chacko *et al.* 1999	1990–1993	NEC requiring laparotomy (N=10)	Gestational age-, birth weight matched controls (N=20)	26wk PCA, ELBW	5 and 7yr	Infants with NEC requiring laparotomy had increased risk of neurodevelopmental problems
Simon *et al.* 1993		NEC requiring laparotomy (N=6)	NEC managed medically (N=12)	Neonatal, VLBW	15mo post term, 24mo	Higher prevalence of motor delays early after surgery; no difference detected at 2yr of age
Miller *et al.* 1995	1987–1989	Open heart surgery (N=91)	None	Neonatal	>2yr	Cerebral palsy in 22%, mean IQ 90, but highly dependent on type of congenital heart disease
Karl *et al.* 2004	1988–1994	ASO with limited DHCA (N=74)	Best friend’ control group or general population (N=74)	0–118 months (median 9)	9.1 +/- 2.9yr	Lower IQ and higher prevalence of behavioural, language expression and comprehension problems than control
Eishima 1992		Intrauterine exposure to nitrous oxide (N=159)	No intrauterine exposure	Prenatal, third trimester	5d postnatal	Weaker habituation to sound, stronger muscular tension and resistance to cuddle, fewer smiles
Blair *et al.* 1984		General or local anaesthetics (N=9)	No anaesthetic exposure (N=30)	Prenatal: first to third trimester	0.8–6d postnatally	Prolongation of visual-pattern preference
Hollenback *et al.* 1986		General or local anaesthetic (N=7)	No anaesthetic exposure (N=7)	Prenatal: first to third trimester	4 +/- 0.008yr	Lower IQ scores
Hollmen 1978		Thiopental, nitrous oxide for general anaesthesia (N=15)	Lignocaine 1.5% for epidural analgesia (N=15)	Perinatal for caesarean section	1–7d	Abnormal neurological activity for up to 7d in 47% regardless of group assignment

**Table 2.** List of included studies.

Reference	Year of birth of cohort	Source of data	Study group	Control group	Age during neurological assessment	Neurological assessment tool	Neurological sequelae in the study group	Strength and weakness	Hazards Ratio (HR)/adjusted Risk Ratio (aRR)
Wilder *et al.* 2009	1976–1982	Birth cohort of children born in Rochester, Minnesota. Medical charts of children were reviewed. Identification of learning disabilities was based on state-wide comprehensive educational records	General anaesthesia before 4 yr (N=593)	No anaesthetic exposure (N=4782)	From surgery till LD criteria met or age 19y	**Combination of individually administered and group administered tests:** LD (based on individually administered intelligence quotient (IQ, primarily age-appropriate Wechsler scales) and achievement (primarily Woodcock-Johnson tests) tests	Exposure to multiple, but not single, anesthetic/surgery significantly increased the risk of developing LDs	Anaesthesia chart of study group were reviewed. Outcome measure was based on comprehensive individual and group test	One exposure HR 1.0 (95% CI 0.79–1.27); 2 exposures HR 1.59 (95% CI 1.06–2.37); >3 exposures HR 2.6 (95% CI 1.6–4.24)
Sprung *et al.* 2009	As above	As above	GA for CS (N=193)	RA for CS (N=304); VD (N=4823)	From birth till LD criteria met or age 19y	As above	No difference in incidence of LD between children whose mother had caesarean section under general anaesthesia or regional anaesthesia, or children who delivered vaginally	As above	LD similar in children delivered vaginally or LSCS with GA but is reduced in LSCS with RA HR 0.64 (95% CI 0.44, 0.92)
Flick *et al.* 2011	As above	As above	General anaesthesia before 2y (N=350)	No anaesthetic exposure before 2y (N=700)	From surgery till LD criteria met or age 19y	As above	Exposure to multiple, but not single, anesthetic/surgery significantly increased the risk of developing LDs	As above	Multiple exposure HR 2.12 (95% CI 1.26, 3.54)
Sprung *et al.* 2012	As above	As above	General anaesthesia before 2y (N=350)	No anaesthetic exposure before 2y (N=5007)	From surgery till ADHD criteria met or age 19y	**Stringent research criteria:** Patients were defined as having research-identified “definite” ADHD if their records included a clinical diagnosis of ADHD and at least 1 form of supporting evidence	Exposure to multiple, but not single, procedures requiring general anesthesia was associated with an increased risk for ADHD	Anaesthesia chart of study group were reviewed. Outcome measure was based stringent criteria	Multiple exposure HR 1.95 (95% CI 6.8, 8.4) vs single exposure HR 1.18 (95% CI 0.79–1.77)
Ing *et al.* 2012	1989–1992	Western Australian Pregnancy Cohort (Raine) Study, an established birth cohort consisting of 2868 children created to evaluate the long-term effects of prenatal ultrasound, of which 2608 were evaluated in this study	General surgery before 3 (N=321)	No anaesthetic exposure (N=2287)	10y	**Individually administered tests:** Comprehensive neuropsychological assessment covering domains on language, cognition, behaviour and motor function	Exposed children had increased risk of disability in language and cognition, even with single exposure, but there was no association with anaesthetic exposure and behavioural problems or motor function deficits	Evaluation of the Raine cohort found differences even after a single exposure. This may be due to the use of more sensitive and comprehensive neurocognitive measures in this cohort. However medical information was based on parents' diary on medical history at regular follow up	Language CELF-R aRR 1.87 (95% CI 1.2–2.93); CELF-E aRR 1.72 (95% CI 1.12–2.64) Cognition CPM aRR 1.69 (95% CI 1.13–2.53). Increased aRR is found even on single exposure in both cognition and language CELF-R aRR 2.41 (95% CI 1.4–4.17); CPM aRR 1.73 (95% CI 1.04–2.83)
Block *et al.* 2012	~1988–2000	Department of Anaesthesia billing records were searched for patients who had one or more of the three groups of operations during infancy and who were between 7.0 and 17.9 yr old on the date of the search (January 28, 2008)	General anaesthesia before 1 yr for hernia/orchidopexy, pylorotomy or circumcision (N=287)	General population	7–17.9 y	**Group administered achievement test:** Composite score of Iowa Tests of Basic Skills and Educational Development	Significantly more children underwent anaesthesia and surgery during infancy had very low test scores (below the 5th percentile), both in our overall sample and the subgroup of 58 patients without CNS problems/potential risk factors. There was also a significant association between scores and duration of anaesthesia and surgery in this subgroup	Relatively coarse outcome measure. Small sample size	N/A
Hansen *et al.* 2011	1986–1990	Cohort was identified from Danish National hospital registry. The outcome was ninth grade test average and average teacher rating	Inguinal hernia repair in infancy (N=2689)	Randomly selected age-matched 5% population sample (N=14575)	Ninth grade	**Group administered test:** Ninth grade test average and average teacher rating	No evidence that relatively brief anaesthetic exposure with hernia repair in infancy reduced academic performance at age 15 or 16y after adjusting for known confounding factors	Large sample size; however, relatively coarse outcome measure. Medical records of randomly selected control group not reviewed	N/A
Bartels *et al.* 2009	1986–1995	1143 twin pairs from the Young- Netherlands Twin Register, but only small number of discordant twin pair were used for analysis. Educational achievement score (formal academic assessment in Netherlands) at age 12 was collected from teachers. Cognitive problems were identified by teacher rating scale	Anaesthesia exposure before age 3 yrs (N=130)	Monozygotic co-twin with no exposure before age 3 yrs (N=130)	12y	**Group administered test:** Scores of Dutch CITO - elementary test and Cognitive problems/Inattention subscale of Conner's teacher Rating Scale	No difference in twins discordant for anaesthesia exposure before 36mo	Relatively coarse outcome measure. Small sample size	N/A
DiMaggio *et al.* 2009	1999–2002	Cohort of children who were enrollees of the New York State Medicaid program - a government funded health insurance program	Inguinal hernia repair before 3y (N=383)	No hernia repair before 3y (N=5050)	From birth to first outcome diagnosis, to last day of year to which child is lost to follow-up or end of study period	**Diagnostic codes identified from administrative database:** Developmental delay and behavioural disorders using ICD-9 diagnosis codes	Hernia surgery before 3 years of age was associated with increased risks for developmental delay and behavioural disorders	Relatively coarse outcome measure, Data retrieved from this program is administrative data and is subjected to measurement errors. Medical and educational records were not reviewed	Hernia repair at <3y aRR 2.3 (95% CI 1.3, 4.1)
DiMaggio *et al.* 2011	1999–2005	As above	Surgery/anesthetic exposure before 3y (N=304)	No exposure before 3y (N=10146)	As above	As above	Repeated exposure to anaesthesia, not single exposure, before age 3y is associated with an increased risk of behavioural or developmental problems	As above	HR of developmental/behavioural disorders with any exposure to anaesthesia <3y is about 1.6 (95% CI 1.4, 1.8). Risk is increased from 1.1 (95% CI 0.8, 1.4) for one operation, 2.9 (94% CI 2.5, 3.1) for 2 operations to 4.0 (95% CI 3.5, 4.5) for more than 3 operations
Kalkman *et al.* 2009	As above	Children underwent pediatric urological surgery in the years 1987, 1991, 1993, 1995 in a pediatric hospital	Urological procedure before 2 yr of age (N=178)	Urological procedure after 2 yr of age (N=65)	11–14y post surgery	**Subjective parental rating:** Parental Child Behavioural Checklist	Although there is a trend towards more atypical behaviour in children with exposure before 2 years of age, it has very wide confidence interval and is not statistically significant. However study is significantly underpowered	Outcome of cognitive assessment was not done by academic source or other formal assessment, but was identified by parental child behavioural checklist. Very small sample size. Medical records not reviewed, it was not uncertain if cohort has single or multiple exposure, or other surgical exposure	N/A

ADQC= Abbreviated Depression Questionnaire for Children Self Assessment; ASO= Arterial Switch Operation; BSID= Bayley Scales of Infant Development; CBCL= Achenbach Child Behaviour Checklist Parental Assessment; CELF-R= Clinical Evaluation of Language Fundamentals-Revised; DDST= Denver Developmental Screening Test; DHCA= Deep Hypothermic Circulatory Arrest; ELBW= Extremely Low Birth Weight (<1000g); GMDS= Griffiths Mental Development Scales; INFANIB= Infant Neurological International Battery; LF-CPB= Low Flow-Cardiopulmonary Bypass; MDI Bayley= Mental Development Index; Movement ABC= Movement Assessment Battery for Children; NDI= Neurodevelopmental Impairment; PDI= Bayley Psychomotor Developmental Index; PDMS= Peabody Developmental Motor Scales; PHBQ= Vernon Post Hospitalisation Behaviour Questionnaire; PPVT= Peabody Picture Vocabulary Test; SBIS= Stanford-Binet Intelligence Scales; TOVA= Test of Variables of Attention; TRF= Teacher’s Report Form; VMI= Developmental Test of Motor Integration; WCST= Wisconsin Card Sorting Test of problem solving; WJPB= Woodcock-Johnson Psychoeducational Battery; VLBW= Very Low Birth Weight (<1500g); WIAT= Wechsler Individual Achievement Test; WISC 3= Wechsler Intelligence Scale for Children, Third Edition; WISC-RN= Wechsler Intelligence Scale for Children; WPPS-R= Revised Wechsler Preschool and Primary Scales for Intelligence.

A group at the Mayo Clinic in Rochester was responsible for four of the included studies, using a birth cohort of children born in Rochester, Minnesota, USA, between 1976 and 1982 ^13– 16^. In one of the investigations ^13^, 593 children with anaesthetic exposure before the age of 4 were compared with 4764 children with no anaesthetic exposure. Children receiving two, three or more anaesthetics were respectively 1.59 or 2.6 times more likely to have subsequent learning disabilities. Using the data from the same cohort, 350 children with anaesthetic exposure were compared with 5007 children with no anaesthetic exposure before the age of 2 years ^16^. Children who had two or more exposures were 1.95 times more likely to be diagnosed with Attention Deficit Hyperactive Disorder (ADHD) than the unexposed children. From the same cohort in a matched design study, 350 children who had anaesthetic exposure before the age of 2 for were compared to 700 unexposed matched controls on the basis of known risk factors for learning disability ^14^. Again children who had two or more anaesthetic exposures, but not single exposure, had an increased risk of subsequent learning disability (hazard ratio 2.12). The last study from the same cohort revealed that a single perinatal exposure to general anaesthesia during delivery by Caesarean section was not associated with an increased risk of learning disability ^15^.

Using another national registry, investigators at Odense University in Denmark identified a cohort of 2689 children born between 1986 and 1990 that had a hernia repair before one year of age ^12^. These children were compared with a randomly selected, age matched population consisting of 14575 children. The average test scores at ninth grade and test score non-attainment rate were used as marker for learning disability. After adjusting for confounding factors, no significant differences in either parameter between the two groups were found.

Investigators at Columbia University used the New York State Medicaid registry to identify a birth cohort of children who had surgery before the age of 3 years ^7, 9^. Medicaid is a health insurance program provided by US government covering approximately 25% of all children in the USA. In the first study 383 children whose insurance codes indicated surgery for inguinal hernia before age 3 years were identified ^9^. These children were compared to a cohort of 5050 matched controls. Insurance codes were used to identify children with behavioural or development disorders. After controlling for potential confounders, children who had hernia repair before 3 years of age were more than twice as likely as controls to be subsequently diagnosed with developmental or behavioural disorder. Using the same Medicaid registry, a birth cohort of 10450 siblings was identified by the same group of investigators ^7^. Three hundred and four children whose insurance codes indicated surgical procedures before the age of 3 years were compared to 10146 children who had no surgery before the age of 3. Similarly insurance codes were used to identify children with behavioural or development disorders. Children who had surgical exposure before the 3 years of age were 1.6 times more likely to have a subsequent behavioural or development disorder. This same group of investigators also used the Western Australian Pregnancy Cohort, which consisted of 2868 children born between 1989–1992, of which 2608 were assessed to identify 321 children who had surgical procedures before age of 3 years ^10^. Learning ability was assessed by more sensitive and specific neuropsychological tests. These tests include the Symbol Digit Modality Test and Raven’s Colored Progressive Matrices for assessment of cognition, the MaCarron Assessment of Neuromuscular Development for assessment of fine and gross motor control, the Clinical Evaluation of Language Fundamentals for assessment of various aspects of language ability. The Child Behavior Checklist (CBCL) was also used for assessment of behavioural problems. Children with single or multiple anaesthesia exposure were shown to have an increased risk of language and abstract reasoning deficits, but there was no association with anaesthesia exposure and behavioural or motor problems. The adjusted risk ratio (aRR) was 1.87 [95% CI 1.2–2.93] for receptive language, 1.72 [95% CI 1.12–2.64] for expressive language, 2.11 [95% CI 1.42–3.14] for total language, and 1.69 [95% CI 1.13–2.53] for abstract reasoning, a domain of the cognitive test.

Investigators at University Medical Centre Utrecht in Netherlands identified 314 children who had urological procedures under general anaesthesia before the age of 6 years ^8^. Neurobehavioral development was assessed using Child Behaviour Checklist (CBCL) returned by parents. This study revealed no association with behavioural disturbances and anaesthesia exposure. Another study from the Netherlands (Vrije Universiteit Amsterdam) attempted to explore causality of anaesthesia exposure and learning related outcomes by using a monozygotic concordant-discordant design ^11^. The researchers identified 1143 monozygotic twin pairs from the Netherlands Twin Registry. Data on anaesthetic exposure and learning outcomes was based on parental reports and standardized test scores respectively. The authors revealed that children from this cohort who were exposed to anaesthesia before the age of 3 years had significantly lower educational achievement scores and more cognitive problems than the unexposed children. However the un-exposed monozygotic twins did not differ from the exposed co-twin.

## Discussion

Clinical observations dating as far back as 60 years have shown an association between exposure to anaesthesia in children and central nervous system dysfunction ^17^. Numerous animal studies on rodents and even non-human primates have since been performed to investigate why this may occur ^18, 19^. Consistently it has been shown that exposure of the developing mammalian brain to most general anaesthetic drugs causes some degree of neuronal apoptosis and neurodegeneration during critical developmental periods ^5^. General anaesthetics are powerful modulators of neurotransmission via a variety of ligand-gated ion channels. The drugs vary in their pharmacodynamic effects and receptor interaction so, to some extent, it is difficult to generalise but they mostly potentiate the gamma-amino butyric acid (GABA) receptor complex and/or inhibit glutamatergic neurotransmission principally through blockade of the N-methyl d-aspartate (NMDA) receptor ( Table 3) ^20^. Both of these neurotransmitter systems are central in determining the excitation/inhibition activity balance underlying experience-dependent sculpting of developing neural networks during the sensitive time of the neuronal “growth spurt” ^21^. This critical period coincides with intense synaptogenesis in most cortical regions. In humans this period of synaptogenesis occurs between the third trimester of pregnancy and first few years of postnatal life; the most marked increase in synapse number occurring between birth and six months of age ^22^. In humans, there are significant regional differences in the timing of the neuronal growth spurt. The earliest is the primary sensorimotor cortex, which occurs around birth, subsequently the parietal and temporal region (important in language and spatial attention) around 9 months and lastly the prefrontal cortex at 2–3 years ^23^. During normal development, neurons are produced in excess by as much as 50–70% and subsequent neuronal pruning is essential for normal brain structure and function ^7, 24^. The mechanism of anaesthesia-induced cell death is not fully understood. Hence it is uncertain whether anaesthesia-induced apoptosis occurs in cells that are not meant to die i.e. pathological apoptosis, or whether it accelerates the death of cells that are meant to die at a later time i.e. premature physiological apoptosis.

**Table 3.** Putative mechanism of sedatives and general anaesthetics on gamma-aminobutyric acid (GABA _A_), type A; and N-methyl d-aspartate (NMDA) receptors.

Drug	GABA _A_ agonist	NMDA antagonist
Benzodiazepines	+++	-
Ketamine	+	+++
Propofol	++	+
Nitrous oxide	+	+++
Isoflurane	+++	+
Sevoflurane	+++	+

“-” = no known effect; “+” = weak effect; ++ strong effect; +++ very strong effect.

Recent clinical studies may lead one to think this is a significant clinical issue for children undergoing surgery but a closer look at the data however, will reveal that the evidence is far from conclusive. While eight studies revealed a positive association between anaesthetic exposure and neurodevelopmental risk, the other two studies revealed the opposite. However, as studies relevant to this question are, to date, retrospective in nature, they only allowed identification of an association without establishing causality. Although there were large number of emigrates in the Rochester cohort, it did contain more than 5000 children. Complete medical and anaesthetic records were reviewed. Data collected included the type of surgery, type of anaesthetic agents, and number of anaesthetic exposures and duration of anaesthesia. Learning disability was assessed using only educational records. Consistently these studies revealed that single anaesthetic exposure during perinatal period ^15^ or before age of 4 years ^13, 14, 16^ was not associated with increased risk of learning disabilities or ADHD behaviour. Multiple exposures however were associated with significantly increased risk for learning disabilities and ADHD disorder and this may signify a dose-response relationship with a progressively increasing risk following two or more operations. Similarly data from the Danish Cohort may indicate that a single brief anaesthetic exposure is not associated with an increased risk of learning disability. The authors using the Western Australia cohort suggested that association between anaesthesia and neurodevelopmental outcome may be confined to specific domains (language and cognition) and this investigation may help to guide future studies.

In four of the investigations where a large cohort of children was identified ^7, 9, 10, 12^, information on surgical exposure and behavioural and development diagnosis were based solely on the administrative data of government funded health insurance program or parent questionnaires. Medical and anaesthetic or educational records were not reviewed and therefore misclassification is possible and therefore results drawn from these data are subjected to measurement error. Moreover the children from the Medicaid registry could be children with disadvantaged background and these results may not be generalized to other population groups. Nevertheless some of the imprecision should only lead to an under-estimation of true association. In the study by Kalkman *et al.* ^8^ the authors commented that this study may be underpowered to reveal any significant differences. Moreover the CBCL may be an insensitive tool to detect neurodevelopment disability. This result is consistent with the finding from investigators at Columbia University as they have shown anaesthetic exposure is not associated with behavioural or motor disabilities ^10^.

In the study involving twins ^11^, the authors concluded that anaesthesia exposure does not cause later learning-related disability. Only a small number of twin pairs were discordant for surgical exposure (130 pairs) and an even smaller numbers of twin pairs had an educational achievement score (110) and a cognitive problem scale (56) available ^25^. Therefore the lack of difference in scores may be secondary to inadequate sample size. Moreover the number of anaesthetic exposures in this cohort was not stated and this could potentially affect the outcome of interest.

In summary the available data from various studies including large numbers of children points to a possible association between anaesthetic exposure in early childhood and learning disability. Moreover a dose-response effect may be present. However one must be cautious with the conclusions drawn from retrospective studies. Association between early anaesthesia exposure and subsequent learning disability does not indicate anaesthesia neurotoxicity. There are many known and unknown confounding factors. Known co-existing medical or surgical diseases and disruption to learning due to repeated hospitalization are examples of such confounding factors. It is not possible to delineate the effect of surgical exposure and hospitalization from anaesthetic exposure. Retrospective data is subject to imprecision or error. The cohorts represented in these studies were children who had anaesthesia and surgery two to three decades ago, there have been many advances in surgical approach and anaesthetic techniques, and hence results from previous treatment may not apply today. Neurocognitive outcome is difficult to study in children and because of the growing complexity in their neurocognitive development as they age. This warrants more types of psychometric tests to assess domains which were not applicable at a younger age, which is to say that more domains need to be tested. Adding to this problem is the fact that it is not known which domain is affected most by anaesthesia related neurotoxicity. Coarse scoring systems such as IQ or measures such as diagnosis of developmental delay may overlook any subtle effects confined to specific areas; however, more refined psychometric tests have an increased chance of finding at least one association purely by chance. Tests carried out at an early age will only uncover major neurological problems and psychometric tests carried out in young children are poor at predicting later outcome ^26, 27^.

Given the limitations inherent in retrospective studies, prospective randomized studies are clearly needed to clarify long-term cognitive effects of early anaesthetic exposure in humans. The main problem is one of confounding factors. The effects of anaesthesia cannot be dissociated from factors associated with anaesthesia, such as surgical trauma and pathology. Surgery is associated with other confounding factors such as humoral and inflammatory stress as well as metabolic, haemodynamic and respiratory events, which may all influence outcomes. Infants and children having surgery or diagnostic procedures are very likely to have pathology, which may influence neurobehavioural outcome. They may be septic, premature, have less parental interaction or have chromosomal abnormalities, all of which can also be associated with developmental delay and need for surgery.

Currently there are two large-scale studies underway that are trying to address the issue of anaesthetic neurotoxicity in children. One that will attempt to separate the effects of general anaesthesia from surgical procedure is the General Anaesthesia Study (GAS) ^28^. This is a multi-centre randomised controlled trial involving 29 centres around the world. The primary objective of this study is to compare regional and general anaesthesia for effects on neurodevelopmental outcome and apnoea in infants requiring inguinal hernia repair. Six hundred infants below 60 weeks post-conception age are randomised to receive either general anaesthesia with sevoflurane or spinal anaesthesia without sedation. The follow-up period will be at 5 years, with evaluation performed at 2 years using the Bayley Scales for Infant Development-III and at 5 years using the Wechsler Preschool and Primary Scale of Intelligence-III and additional neuropsychological tests within NEPSY-II (A developmental NEuroPSYchological assessment). The expected date of completion is 2015/2016. The other one is the PANDA (Pediatric Anaesthesia and Neurodevelopmental Assessment) study, which is another multi-centre study that involves eight US sites. This study proposes using a bidirectional epidemiological approach where a historical cohort exposed to a single general anaesthesia for inguinal hernia repair American Society of anaesthesiologist (ASA) class 1 and 2 before 36 months of age is identified. The group will be followed up prospectively using neurocognitive testing between the ages of 6 and 10 years. This study is an attempt to reduce the genetic and environmental contributions to cognitive performance. The pilot study has been completed, which demonstrated feasibility of such an approach ^29^.

Therefore, it is clear from preclinical data that anaesthetic agents are associated with neurotoxicity in developing animals ^30, 31^. However interpretation of clinical studies that have been completed to date is less clear-cut. This is due to the retrospective nature of the studies, the lack of specific information in terms of age, duration, and dose of anaesthetics, precise agents used, the variable outcome endpoints used and the way these outcomes were assessed. Many of the outcome measures were lacking in specificity and standardization in most cases. Any change in anaesthetic practice should be evidence based. The Anaesthetic and Life Support Drugs Committee of the U.S. Food and Drug Administration held a meeting in March 2011 and concluded that they acknowledge the compelling animal data that anaesthesia exposure is neurotoxic to the developing brain. However, there is still not enough data, especially in humans, to draw any firm conclusions.

## Conclusions

In conclusion although there are some data suggesting a possible detrimental effect of anaesthesia on the developing brain in children, the evidence is best considered preliminary and inconclusive at this stage. However what we do know is that it is unethical to subject infants and children to surgery without the benefits of anaesthesia and analgesia. Parents should be counselled to not avoid necessary invasive procedures for fear of a currently ill-defined risk.
