# Supplementary material for: The effects of anaesthesia on the developing brain: a summary of the clinical evidence
Source: F1000Res. 2013 Aug 20;2:166. Originally published 2013 Aug 2. [Version 2] doi: 10.12688/f1000research.2-166.v2 (PMC3829131; doi:10.12688/f1000research.2-166.v2)
Supplement: Peer review history

# Peer review: Referee response for version 2

**Published:** 2013-08-30

**Authors:** Caleb Ing

**Affiliations:** 1 Pediatric Anesthesia, Columbia University Medical Centre, New York, 10032, USA

The revised version has addressed the identified corrections.

I have read this submission. I believe that I have an appropriate level of expertise to confirm that it is of an acceptable scientific standard.

# Peer review: Referee response for version 2

**Published:** 2013-08-28

**Authors:** Igor Luginbuehl

**Affiliations:** 1 Univeristy of Toronto, Toronto, Canada

This updated version corrects and specifies the findings of one study in particular. The overall message/conclusion of the review however remains unchanged, which I think is a good thing.

I have read this submission. I believe that I have an appropriate level of expertise to confirm that it is of an acceptable scientific standard.

# Peer review: Referee response for version 1

**Published:** 2013-08-07

**Authors:** Igor Luginbuehl

**Affiliations:** 1 Univeristy of Toronto, Toronto, Canada

This is a very complex topic of significant importance and interest not only to anesthesiologists, but also to parents and, of course, the patients. A lot of research has been done in that regard, but we are still awaiting a definite answer, which will most likely take a long time.

I have read this submission. I believe that I have an appropriate level of expertise to confirm that it is of an acceptable scientific standard.

# Peer review: Referee response for version 1

**Published:** 2013-08-06

**Authors:** Caleb Ing

**Affiliations:** 1 Pediatric Anesthesia, Columbia University Medical Centre, New York, 10032, USA

This paper presents a nice summary of the clinical studies that have been performed to evaluate the long-term effects of anesthetic exposure in young children. However, in order to improve the accuracy of the paper, there were a few corrections to point out:

- The Western Australian Pregnancy Cohort Study (Raine) consisted of 2868 children of which 2608 were evaluated in the cited study, with 321 who had surgical procedures before age 3 years.

- Table 2: In the Raine Cohort, the study group was composed of 321 children exposed to anesthesia and 2287 children who were unexposed.  The number of children tested for each outcome however varied by individual outcome and also varied during the evaluation of single and multiple anesthetic exposures.  While several studies only found a difference in cognitive outcomes after multiple anesthetic exposures, evaluation of the Raine cohort found differences even after a single exposure.  This may be due to the use of more sensitive directly assessed neurocognitive measures in this cohort. 

Also as a clarification, in this study, the age of neurological assessment for all children was at age 10.

I have read this submission. I believe that I have an appropriate level of expertise to confirm that it is of an acceptable scientific standard.
